# 

**Published:** 1856-10

**Authors:** 


### BOOKS RECEIVED.

Barclay (A. Whyte, M.D.) The Progress of Preventive Medicine and Sanitary Measures. London: Bell and Daldy. 1856.

Barker (T. Herbert, M.D.) On the Relative Value of the Ozonometers of Drs. Schönbein and Moffat. London: 1856.

Barnes (Robert, M.D.) Report of the Sanitary Condition of Shoreditch for the Quarter ending June 30th, 1856. London: 1856.

Census of Ireland for 1851. Parts V and VI. Dublin: 1856.

Dickens (A. L., C. E.) Report of the General Board of Health on an Inquiry with respect to the Proceedings of the Local Board of Health of Sandgate. London: 1856.

Glaisher (James, F.R.S.) On the Meteorology of England and Scotland during the Quarters ending March 31 and June 30, 1856. London: 1856.

Houghton (J. H.) Sixth Quarterly Report on the Sanitary State of Dudley. Dudley: 1856.

Lindsay (W. Lauder, M.D.) Histology of the Cholera Evacuations in Man and the Lower Animals. Edinburgh: 1856.

Macdonald (J. D.) Anatomy of the Nautilus, reprinted from *Philosophical Transactions*. London: 1856.

Michael (W. H.) Medical Report on the Sickness and Mortality of Swansea. Swansea: 1856.

Moncriff (Bernard.) The Philosophy of the Stomach, or an exclusively Animal Diet the most wholesome and fit for Man. London: Longmans. 1856.

Spence (J.), and Glaisher (J.) On the Similarity of Form in Snow Crystals and Camphor under certain Forms of Crystallisation. London: 1856.

Steele (John Charles, M.D.) Observations on the Construction and Ventilation of Hospitals for the Sick. Glasgow: 1853.

Tripe (John W., M.D.) Report on the Past and Present Sanitary Condition of the Hackney District. London: 1856.

Turnbull (James, M.D.) A Practical Treatise on Disorders of the Stomach with Fermentation. London: Churchill. 1856.

Webster (John, M.D., F.R.S.) Report on Lunacy Laws and Asylums. Pamphlet. London: 1856.

Whitfield (R. G.) The Administration of Medical Relief to the Out-Patients of Hospitals. Pamphlet. London: Barrett. 1856.

---

### IN EXCHANGE.

Scottish Review.
Gazette Médicale de Paris.
Dublin Hospital Gazette.
Literary Gazette.
Glasgow Medical Journal.
Psychological Journal.
Association Medical Journal.
American Journal of the Medical Sciences.
New York Journal of Medicine.
Montreal Medical Chronicle.
Ranking and Radcliffe's Abstract of the Medical Sciences.
Chemist.
Veterinarian.
Charleston Medical Journal.
Edinburgh Medical Journal.
Asylum Journal of Mental Science.
Quarterly Journal of the Chemical Society.
Aerzliches Intelligenz-Blatt.

### NOTES AND CORRESPONDENCE.

### INVISIBLE IMPURITIES OF LONDON.

The third class of atmospheric impurities is invisible, but it arises from the long retention of the excrement of London under the houses and in the sewers. According to the estimate of Mr. Lawes, London could supply the farmers of England daily with 29 tons of ammonia, 51 tons of carbon, 14 tons of phosphates, 32 tons of mineral matter, and 14 tons of other matter, making in the aggregate 140 tons of dry manure, dissolved naturally in about nineteen times its weight of water. The country requires this precious manure.

The problem for the engineer to solve is, How can 3,000 tons of *town guano* be returned daily to the disinfecting soil, from which it was chiefly taken, with the least offence to health, and with the least cost? Shall it be distributed by pipes, or by railways? Shall it be disinfected by water, earth, ashes, or any chemical compound?

Under the present arrangements, some hundreds of thousands of tons of this matter lie in store in London, putrefying in cesspools and percolating in streets; while the residue is thrown into the Thames at great cost.

All these impurities of the air we breathe in London have evidently a natural tendency to increase more rapidly than the population, and can only be removed by the vigilance, intelligence, and energy of the Board of Works.

Every substantial sanitary improvement they effect, will be as evident as the diminution in the mortality of the districts which now receive purer water, and which will, it may be expected, ere long receive it on the system of constant supply. (*Registrar General's Reports*, Aug. 16, 1856.)

---

The Editor is anxious to give free scope to the expression of opinion, but does not hold himself responsible for the opinions of those of his contributors who attach their names to the articles furnished by them.

Papers by Drs. Pickells and P. B. Ayres, by Mr. Lake, and Mr. Rigden, are in hand, and will appear in next number, as will also a short and interesting paper by Dr. Winn.

Thanks are due to many new contributors to the Local Reports of Disease. Papers for recording observations will be sent to any other Gentlemen who may kindly offer their assistance to this department of the Journal.

---

### To New Subscribers to the Journal of Public Health.

The Publisher begs to intimate that new Subscribers wishing to have the series of the Journal complete from the first number, can obtain sets of Vol. I, at Subscriber's prices, 10s. post free, by application to him, including Post-Office Order, payable at the Charing Cross Branch, to Thomas Richards.

The Journal of Public Health is sold by all Booksellers, and is equally suited for the general and professional reader, for the scientific society, and the private library.

London: T. RICHARDS, 37, GREAT QUEEN STREET.

